# Invasive imaging modalities in a spontaneous coronary artery dissection: when “believing is seeing”

**DOI:** 10.3389/fcvm.2023.1270259

**Published:** 2023-10-18

**Authors:** Zlatko Mehmedbegović, Igor Ivanov, Milenko Čanković, Zoran Perišić, Tomislav Kostić, Bojan Maričić, Gordana Krljanac, Branko Beleslin, Svetlana Apostolović

**Affiliations:** ^1^ Faculty of Medicine, University of Belgrade, Belgrade, Serbia; ^2^ Department of Cardiology, University Clinical Center of Serbia, Belgrade, Serbia; ^3^ Faculty of Medicine, University of Novi Sad, Novi Sad, Serbia; ^4^ Cardiology Clinic, Institute for Cardiovascular Diseases of Vojvodina, Sremska Kamenica, Serbia; ^5^ Faculty of Medicine, University of Niš, Niš, Serbia; ^6^ Division of Interventional Cardiology, University Clinical Center Niš, Niš, Serbia

**Keywords:** SCAD, diagnostic algorithm, intravascular imaging, IVUS, OCT

## Abstract

Spontaneous coronary artery dissection (SCAD) is a rare but increasingly recognized cause of acute coronary syndrome (ACS) with recent advancements in cardiac imaging facilitating its identification. However, SCAD is still often misdiagnosed due to the absence of angiographic hallmarks in a significant number of cases, highlighting the importance of meticulous interpretation of angiographic findings and, when necessary, additional usage of intravascular imaging to verify changes in arterial wall integrity and identify specific pathoanatomical features associated with SCAD. Accurate diagnosis of SCAD is crucial, as the optimal management strategies for patients with SCAD differ from those with atherosclerotic coronary disease. Current treatment strategies favor a conservative approach, wherein intervention is reserved for cases with persistent ischemia, patients with high-risk coronary anatomy, or patients with hemodynamic instability. In this paper, we provide a preview of invasive imaging modalities and classical angiographic and intravascular imaging hallmarks that may facilitate proper SCAD diagnosis.

## Introduction

**Figure 2:**
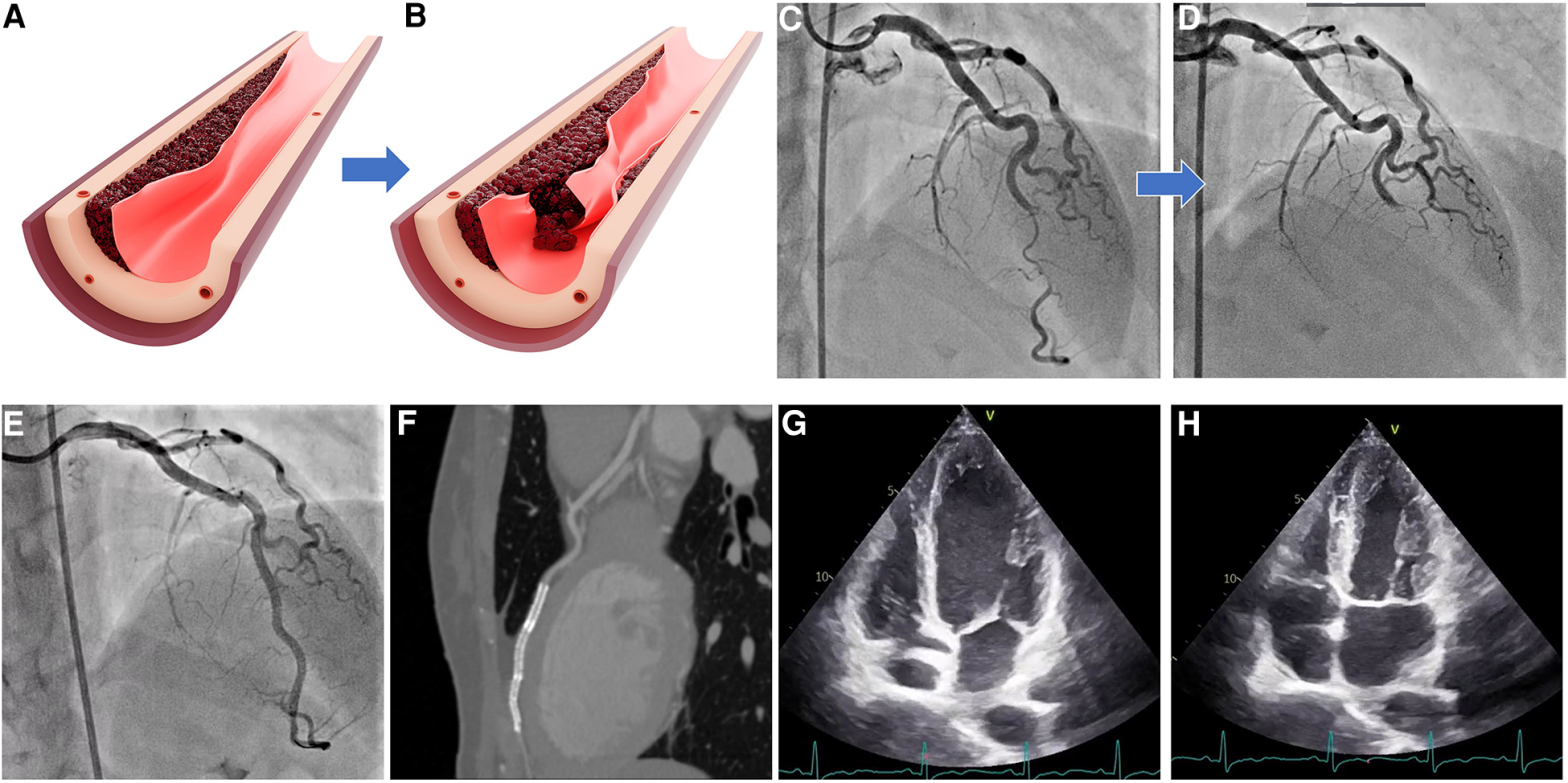
SCAD types 2 and 4. (**A**) Illustration of moderate intramural hematoma without intimal tear; (**B**) propagation of hematoma with tear causing complete vessel occlusion; (**C**) angiographic appearance of SCAD type 2; (**D**) angiographic appearance of SCAD type 4; (**E**) result after three long overlapping stent implantations; (**F**) control MSCT poststenting showing overlapping stents and no evidence of residual dissections; and (**G,H**) echocardiographic evidence of complete restoration of wall contractility after successful stent implantation. SCAD, spontaneous coronary artery dissection.

Spontaneous coronary arterial dissection (SCAD) is widely recognized as one of the causes of acute coronary syndrome (ACS) (1, 2). SCAD starts with the initial formation of a hematoma in the tunica media within the coronary vessel wall (inside-out mechanism) (3). Intramural hematoma may progress distally and circumferentially along the vessel wall compressing the true lumen, resulting in flow disturbances or even complete artery blockage (Figures 2A,C). Hematoma as the initial damage to the vessel wall integrity can subsequently lead to an intimal tear, which gives SCAD its classic angiographic recognition—such as lumen compression, dissection stripes, or a combination of both, and even complete occlusion in some cases (Figures 2B,D). Irrespective of the underlying substrate, the resulting compression of the true lumen clinically produces classical symptoms of ACS. However, the diagnosis of SCAD among patients with ACS might be challenging when relying exclusively on clinical presentation (4). Differential diagnosis of SCAD from common atherothrombotic ACS events is difficult due to the overlapping findings obtained from non-invasive diagnostic modalities such as cardio-specific biomarkers and electrocardiographic or echocardiographic examinations (5). Contemporary non-invasive diagnostic modality for coronary artery visualization, such as multislice computed tomography coronary angiography (MSCT-CA), has several disadvantages, including the fact that it is not commonly employed for streamlining of ACS cases and has a lower spatial resolution, which poses challenges in accurately identifying the pathognomonic features that are crucial for SCAD diagnosis (6). Hence, the diagnosis of SCAD is still mainly based on accurately identifying and interpreting SCAD hallmarks found on invasive coronarography combined and backed-up with pretest probability of a given case (4). Despite the growing appreciation of SCAD angiographic features, the absence of specific hallmarks or angiographic similarities to other possible pathoanatomic substrates (embolus, contrast streaming, myocardial bridging, etc.) leaves significant number of SCAD cases undiagnosed, or in a later scenario, misdiagnosed (7). Therefore, in angiographically ambiguous cases, invasive intravascular modalities can provide valuable information facilitating accurate diagnosis, thereby informing further conservative treatment options or, if needed, guiding to revascularization approach (8). Further to this, SCAD patients, contrary to classic atherothrombotic events caused myocardial infarctions, are managed essentially differently, both in the Cath lab and in the coronary care intensive units (9). PCI in SCAD patients has high complication rates and low angiographic success rates, while the conservative management in majority of cases results in favorable patient outcomes with spontaneous resolution of vessel integrity (1). Thereby, for the same ACS clinical settings, physicians may choose a more conservative approach rather than an intervention-focused treatment approach for SCAD patients during regular primary PCI. In concordance with this, current consensus is that the conservative strategy is a default approach for SCAD cases, whenever it can yield positive outcomes (10). Therefore, optimal SCAD treatment relies on accurate diagnosis, commencing with high level of suspicion combined with unequivocal recognition of traditional SCAD hallmarks by invasive imaging. In this review, we will examine the diagnostic clinical work-up for SCAD, acknowledging the practical aspects of commonly used imaging modalities.

## Pretest probability

The likelihood of SCAD occurring prior to a patient undergoing cardiac catheterization depends on several factors that warrant special attention in a particular case: gender, age, genetic background, clinical presentation, presence of triggering factors, hormonal changes, etc. (11). In most cases of SCAD, patients typically manifest as ACS (1). Most often, biomarkers of myocardial injury are elevated, except perhaps when the presentation is very early, as in ACS (12). Hence, diagnostic doubt should be raised in non-acute cases, but still confirmed among ACS patients. According to registries, SCAD shows gender prevalence for women (13). SCAD in males only occurs approximately 10% of the cases. SCAD accounts for more than one-third of the cases in women under 50 years of age, and up to two-thirds of all pregnancy-associated ACS. Therefore, the index of suspicion of potential SCAD should always be raised in cases involving younger female patients.

According to registries, 90% of SCAD cases have been reported to occur in patients aged 47–53 years (mean 52 years) (14). SCAD is rare in very young (less than 20 years) and very old (above 80 years) adults. Therefore, individuals presenting with symptoms outside this age range should be evaluated more carefully prior to confirming the diagnosis of SCAD; however, diagnostic alertness should not be neglected for older population.

Compared with age and gender, the presence or absence of atherosclerotic risk factors is less useful in predicting the likelihood of SCAD. Although diabetes, hyperlipidemia, and classical risk factors are rarely prediagnosed, the preexisting atherosclerotic disease burden does not exclude the SCAD diagnosis (1, 12). Specifically, hypertension can be present in approximately one-third of the patients with SCAD.

SCAD is associated with a small number of known genetic disorders. Over the past years, significant progress has been made in our understanding of the genetic causes of SCAD. Rare genetic variations, typically in genes linked to hereditary arteriopathies or connective tissue diseases [adult polycystic kidney disease, migraine, fibromuscular dysplasia (FMD), and cervical arterial dissection], are found to be associated with SCAD in up to 50% of the cases (15–17). Recent genetic research suggests that both common and uncommon genetic variables may contribute to the susceptibility to SCAD. Until further evidence, a possible diagnosis of SCAD should be considered in an ACS patient with a family history or clinical features linked to genetic disorders.

The symptoms of SCAD *per se* do not serve as reliable diagnostic differentiators, as they exhibit similarities to symptoms observed in other types of ACS (13). Certain cases have documented potentially provoking stimuli such as emotional or physical stressors. For example, in a particular scenario, if symptoms appear during or after intense isometric training, the likelihood of SCAD diagnosis increases (18). On the other hand, exposure triggers can occur along with other causes of ACS, such as Takotsubo syndrome or during vigorous activity with atherosclerotic plaque rupture. Therefore, patient behavioral factors cannot either confirm or disprove a diagnosis of SCAD but can help the physician fine-tune the level of suspicion of this entity prior to and following the findings of invasive diagnostic tests.

SCAD during pregnancy can pose a significant risk, exposing approximately 1.8% of every 100,000 pregnant women at risk in the United States (19). Available evidence suggests that pregnancy-related physiological changes present risk factors for SCAD such as high progesterone levels and the rapid changes in hormones at birth and during the postpartum period (19, 20). Hormonal background with other stressors and arteriopathies can contribute to the emergence of SCAD, alongside psychological and physical precipitating stressors that have been identified as provoking risk factors. Similar to SCAD, fibromuscular dysplasia affects younger women and is also presently underdiagnosed (16). Because FMD affects the artery walls causing them to lose flexibility and become weak, its greater occurrence in women relative to men implicates estrogen effect, along with the evidence of other hormonal exposures such as fertility treatments, chemical contraception, hormone replacement therapy, and pregnancy, in its emergence (20). Currently, it is not fully understood if these conditions are underlying causes or occur simultaneously with SCAD.

## Angiographic SCAD diagnosis

Currently, coronary angiography (CA) is the primary diagnostic modality for SCAD due to its universal availability (2). If a coronary dissection is suspected, CA should be performed as soon as possible, also in accordance with ACS treatment standards. A reliable diagnosis is crucial because the treatment for these patients differs significantly from that for ACS caused by atherosclerosis. One disadvantage is that it is essentially just a “lumenography” and provides little information regarding the integrity of the artery wall. The extreme coronary (screw-like) tortuosity, preference for the mid-to-distal segments of the vessels, absence of coexisting atherosclerosis, uniform reduction of vessel lumen, strip-like radiolucent filling defects or staining of contrast medium within the arterial wall are pathognomonic angiographic features that may indicate SCAD (21, 22). However, it should be particularly emphasized that because of the SCAD and underlying artery fragility, invasive procedure such as coronarography increases the risk of iatrogenic dissection (approximately 2%–3% risk of iatrogenic dissection is reported vs. 0.2% risk in atherosclerotic patients) (1, 23). Procedure-related dissection in SCAD patients is reported to occur in 14% of patients undergoing PCI. The commonly used classification system is proposed by Saw et al. (21), which includes three distinctive angiographic types (Table 1). The practicality of the proposed classification lies in the ability to suggest further imaging modality to confirm or disprove diagnosis and to indicate treatment, based on SCAD angiographic subtype.

**Table 1:** Angiographic features of SCAD types with intravascular imaging recommendations.

SCAD	Specific diagnosis suggestive angiographic features	Intravascular imaging
Type 1	– Present in about 10%–15% cases – Pathognomonic multiple radiolucent lumen – Contrast dye staining of arterial wall – Presence or absence of dye hang-up or slow contrast clearing from the lumen – Sluggish flow at the within and after dissection segment – Requires intravascular imaging to safely guide treatment	– If needed, may be used to confirm diagnosis and to guide intervention (true lumen wire placement) and optimize result (ensure compression of false lumen) – OCT preferred due to ease of identification of intimal flap (IVUS for experienced imagers) – If used, careful manipulation is needed, since device placement can aggravate the dissection and worsen flow
Type 2	– Most prevalent (60%–75% of the patients) – Typically diffuse >20–30 mm (frequently up to most distal artery segments) – Smooth uniform narrowing (usually moderate in severity) appearing suddenly – Can mimic spasm (not responsive to nitroglycerine) – Usually, no other signs of atherosclerotic involvement	– If needed, may be used to visualize intramural hematoma volume, distribution and longitudinal extension and/or help device selection (cutting balloon, stent) and sizing – On IVUS hematoma is difficult to differentiate from homogenous plaques
Type 3	– Mimics classic atheroma lesions due to its focality – Discrete “sole” lesions (11–20 mm) – Hazy in appearance, mimics intraluminal thrombus	– May be used to visualize intramural hematoma and/or exclude atheroma involvement
Type 4	– Total vessel occlusion – Usually involves a distal vessel segments – Sources of coronary embolism need to be suspected and excluded	– May be used to guide intervention and optimize results and/or help exclude atheroma presence and thrombotic involvement

IVUS, intravascular ultrasound; OCT, optical coherence tomography.

**Figure 1:**
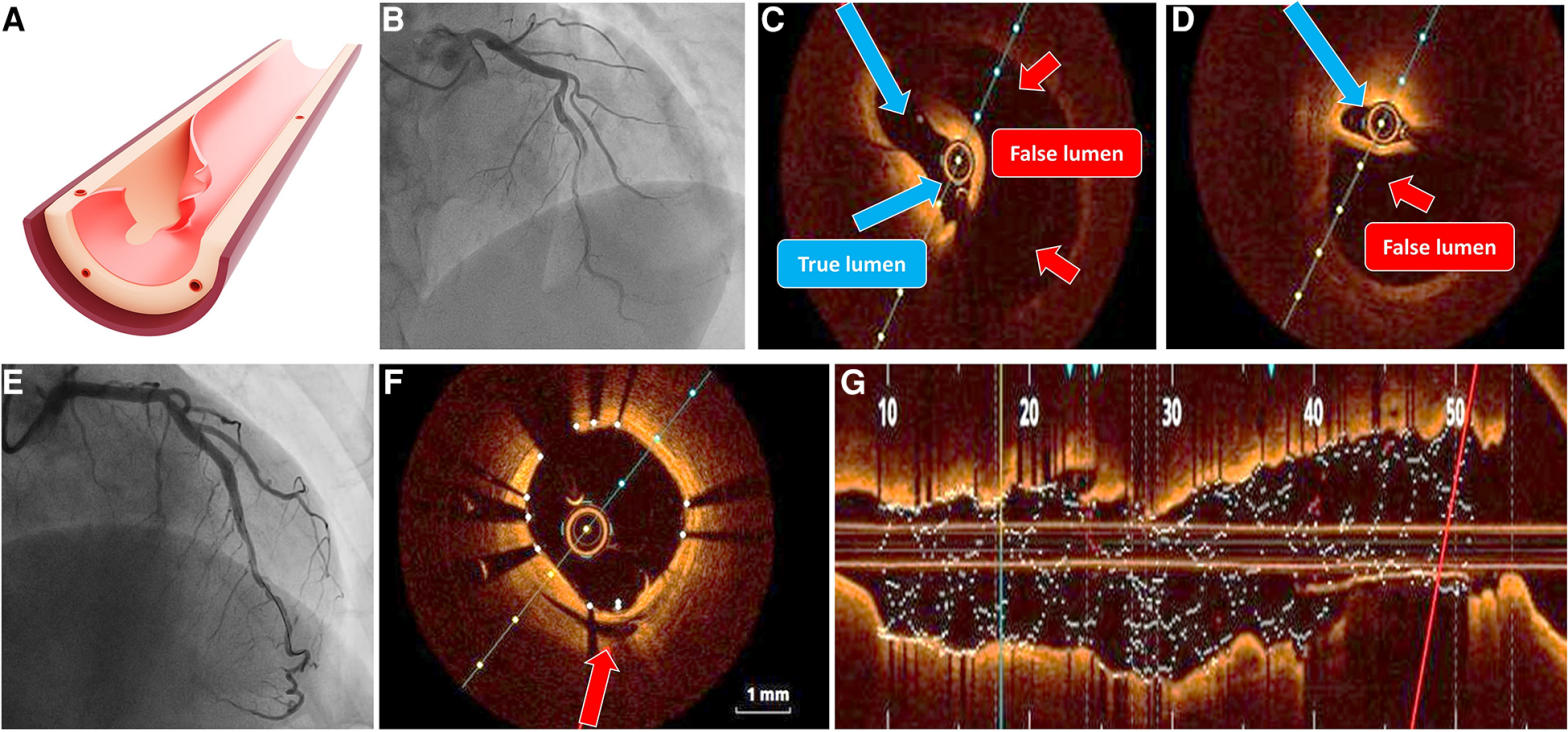
SCAD type 1. (**A**) Illustration of intimal tear; (**B**) angiographic classical radiolucent dissection line along left anterior descending artery; (**C,D**) OCT imaging confirming the presence of double-lumen and intimal flap (true lumen blue arrows, false lumen red arrows) without evidence of atherosclerosis or thrombosis; (**E**) result after long stent implantation; (**F**) OCT cross section showing good stent apposition with compression of false lumen (green arrow); and (**G**) longitudinal OCT image after optimal stent implantation, without evidence of residual dissection. OCT, optical coherence tomography.

The underlying disruption of the artery wall mainly or solely consists of intimal tear, spreading longitudinally and/or circumferentially representing anatomical substrate of SCAD type 1. Because intimal tear allows the contrast dye to enter through two flow channels, type 1 SCAD has the pathognomonic angiographic appearance of an arterial dissection, including multiple radiolucent lumens divided by radiolucent flap (Figure 1B).

In addition to the visible flap and the compartmentalization of the lumen, using of contrast material may result in slow clearance, hung-up, and persistent staining, with or without accompanying flow disturbance. If needed, disrupted intima can be easily visualized with optical coherence tomography (OCT), but the risk of dissection propagation during wire and catheter manipulation, as well as vigorous dye injection, must be thoroughly assessed to evaluate the expected advantages of achieving an unequivocal diagnosis.

SCAD type 2 is the most prevalent type, occurring in approximately 70% of cases. The characteristic angiographic appearance often exhibits a smooth, uniform, tubular structure with a sudden reduction in the lumen diameter (Figures 2A,C). The underlying substrate is compression of the true lumen by an intramural hematoma that develops suddenly and propagates distally along the vessel wall, exerting an extrinsic pressurized effect on the true lumen while leaving the intimal border intact. Type IIa lesions affect the short, localized segment of the vessel, while type IIb lesions show complete distal vessel involvement. Differential diagnosis compromises classic atherosclerotic plaques; therefore, the probable diagnosis of SCAD type 2 should be considered in cases when there are no other atherosclerotic lesions, or when such instances occur within tortuous segments. In addition, intravascular imaging should be reserved for ambiguous cases, particularly considering that conservative treatment is recommended in majority of cases.

**Figure 3:**
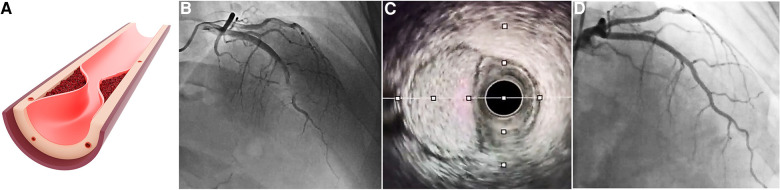
SCAD type 3. (**A**) Illustration of discrete short hematoma presence mimicking classic lesion; (**B**) angiographic appearance of SCAD type 3; (**C**) IVUS imaging showing large intramural hematoma with preserved “uncompressed” lumen; and (**D**) control angiography after hematoma resolution following conservative approach. IVUS, intravascular ultrasound; SCAD, spontaneous coronary artery dissection.

If hematoma localizes in short segments (less than 20 mm), it can mimic short atherosclerotic lesions. This presents the substrate of typical type 3 SCAD. Due to extreme angiographic similarity to atherosclerotic lesion, intracoronary imaging is frequently required to make the precise diagnosis (Figure 3C).

SCAD type 4, newly proposed by Al-Husseini et al., compromises complete occlusion of the vessel (24). Since this is a common finding in regular ACS patients, this type presents significant challenges to be diagnosed without uncertainty. Since vessel occlusion needs to be resolved, as in classical ACS, precise diagnosis should be subsided to optimizing flow and patient patency. Alternative diagnosis can also be suspected such as thromboembolic occlusion (Figures 2B,D).

Importantly, although SCAD hallmark is a “sole” lesion, vessel fragility is an unlocalized feature, and simultaneous multivessel dissections can occur in approximately 10% of cases (25, 26).

## Intravascular SCAD imaging modalities

**Figure 4:**
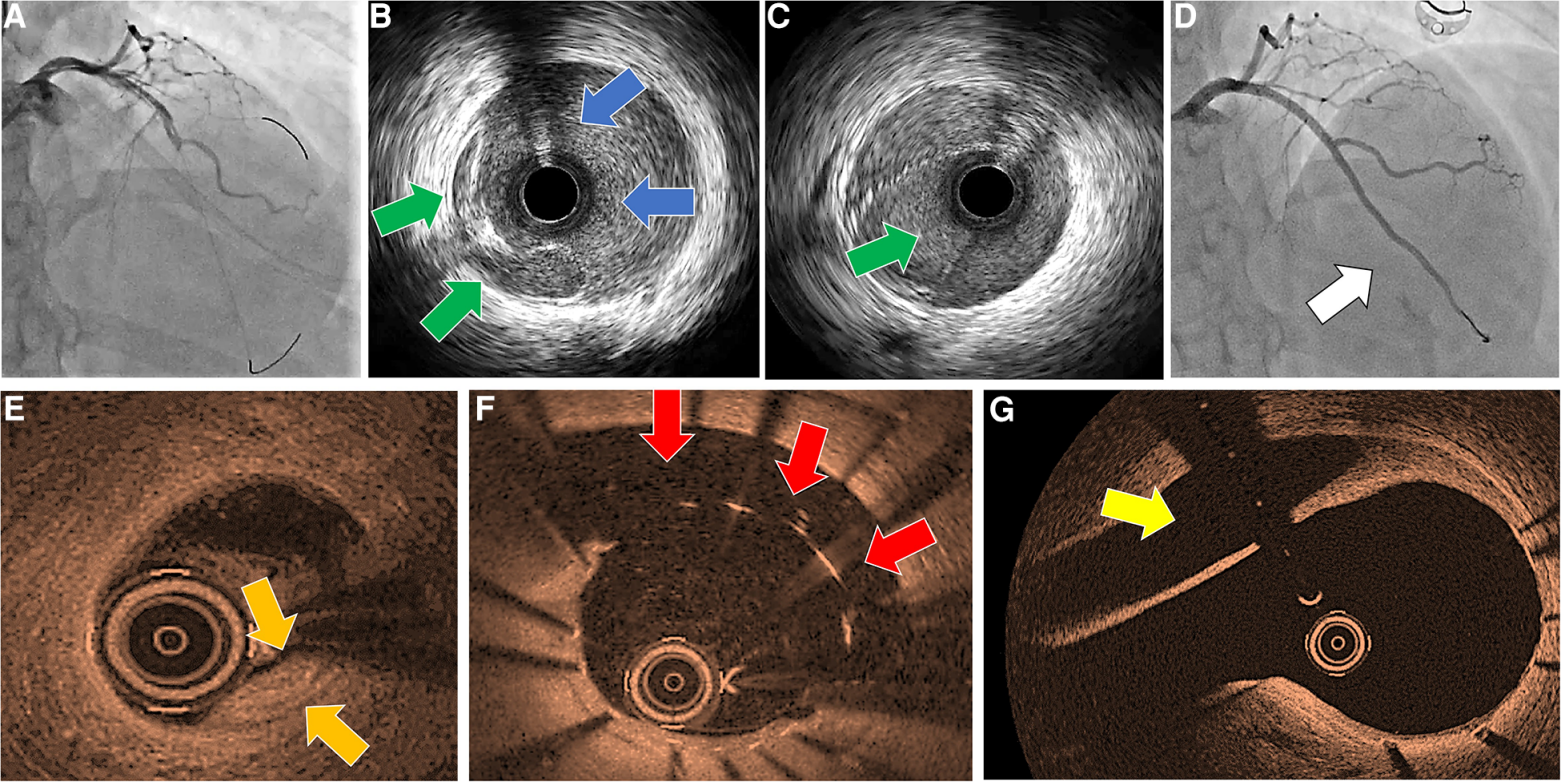
Iatrogenic dissection in an SCAD patient during invasive imaging. (**A**) First angiographic scene after catheter caused dissection of left-main artery showing distal wide-spreading of dissection; (**B**) IVUS probe in false lumen (blue arrows), showing compressed true lumen (green arrows); (**C**) IVUS probe in true lumen (green arrows); (**D**) result after overlapping stent implantations form left-main to distal left anterior descending; (**E**) control in-hospital OCT imaging showing compression of true lumen in distal artery (initial SCAD provoking substrate, compressed dissected lumen, orange arrows); (**F**) stent malapposition with persisting large dissection lumen (red arrows); (**G**) dissection lumen in left-main artery, caused by catheter engagement (yellow arrow). IVUS, intravascular ultrasound; OCT, optical coherence tomography.

Both contemporary invasive imaging modalities, OCT and IVUS, are capable of providing detailed phenomena that are characteristic of SCAD type lesions, such as existence of intimal flap, presence and length of extension of intramural hematoma, possible presence of intramural thrombus, absence of classical atherosclerosis (2, 26, 27). Thus, in addition to possessing unquestionable diagnostic value, they can also assist us in implementing the best PCI strategy (choosing the stent length, cutting balloon diameter, geographic landing location to cover the entry and/or exit site of the dissection, etc.) (28). Before deciding to use these intravascular devices, it is necessary to take into account the potential risks: extension of the dissection by the subintimal placement of the guide catheter, wire, or device itself, hydraulic extension of the false lumen with the application of contrast in the case of OCT, further unwanted compromise of flow due to a small residual circulating lumen when devices are in place, iatrogenic dissection with a catheter during manipulation (common when SCAD is associated with connective tissue weakness), etc. (Figure 4) (23). Therefore, imaging methods should only be used if the artery's lumen is sufficiently large, if angiographic findings are ambiguous, and/or if a further PCI approach has been determined. High operator attentiveness and clearly defined angiographic varieties of SCAD lesions help to avoid the unnecessary utilization of these modalities while preserving the degree of readiness for their rational use.

Intravascular ultrasound provides grayscale images of coronary arteries and walls by a catheter tipped with ultrasound probe (29, 30). One of the advantages of IVUS imaging is its wide availability. In addition, IVUS imaging does not require the application of contrast dye and allows sufficient circumferential field depth for visualization of even large vessels. As such, IVUS can provide valuable information regarding the proportions of the false lumen and the extent of hematoma and can show the false-true lumen separation. However, its limited spatial resolution (100–150 µM) and insufficient grayscale discrimination between homogenous areas (such as hematoma and lipid-rich atherosclerotic plaques) can result in undesirable diagnostic uncertainty, particularly when used by less experienced operators.

Optical coherence tomography is an intravascular imaging modality that uses infrared light technology to produce images with 10 times higher resolution than IVUS (29, 30). Instead of using ultrasound, the method employed near infrared light technology, which is absorbed and reflected by tissues and structures upon interaction, depending on their composition. Tissue characterization is accomplished by digital interpretation of the intensified or attenuated recaptured optical signals, even allowing precise intimal border visualization. Therefore, it is the most sensitive modality to depict false-true lumen and intramural hematomas, essential for confirming SCAD diagnosis. However, since it requires vigorous contrast dye injections, it possesses great risk for dissection enlargement. In addition, its use in real-time guide wire manipulations (when true lumen wire negotiation is attempted) is limited compared with IVUS (Figures 4A,B) since it has a short mode of image recording of just approximately 2 s in the automatic pullback mode.

## Non-invasive SCAD imaging modalities

Other imaging modalities, such as MSCT-CA, echocardiography, myocardial perfusion imaging (MPI), or cardiac magnetic resonance (CMR), can provide additional diagnostic information for streamlining the possible SCAD cases that can lead to coronarography and exact diagnosis (28, 31). For instance, the presence of myocardial ischemia or infarction as detected by regional wall motion abnormalities using focused echocardiography, the decreased myocardial perfusion on MPI, or the detection of myocardial infarction by late gadolinium enhancement detected by CMR can provide complementary evidence and support the diagnosis (32). According to literature, CMR can be considered in cases when SCAD is suspected to confirm the occurrence of myocardial infarction, to assess the extent of myocardial involvement, and more importantly to elucidate concurrent etiologies and sequelae (33).

The MSCT-CA is commonly employed as the initial assessment tool for low-risk patients or clinically uncertain cases, since it is widely adopted by medical facilities for non-invasive imaging in the first-line assessment of ACS patients. The advancements in spatial and temporal resolution have greatly enhanced the ability to assess the main epicardial vessels. However, the current MSCT-CA still lacks the capability to accurately assess small distal coronary arteries due to its limited resolution. There are several distinct features that can be seen on MSCT-CA that can help or even confirm the diagnosis of SCAD, including the absence of atherosclerotic plaque, tapered luminal stenosis, abrupt luminal stenosis, luminal occlusion, intramural hematoma with hemorrhage within the wall of the coronary artery, dissection flap, and perivascular epicardial fat stranding. In addition, the likelihood of SCAD can be increased in cases when there is cardiac hypoperfusion occurring in a similar vascular area. However, such findings are not specific to SCAD and are frequently observed in other acute coronary syndromes (34).

## Holistic SCAD diagnostic pathway

**Figure 5:**
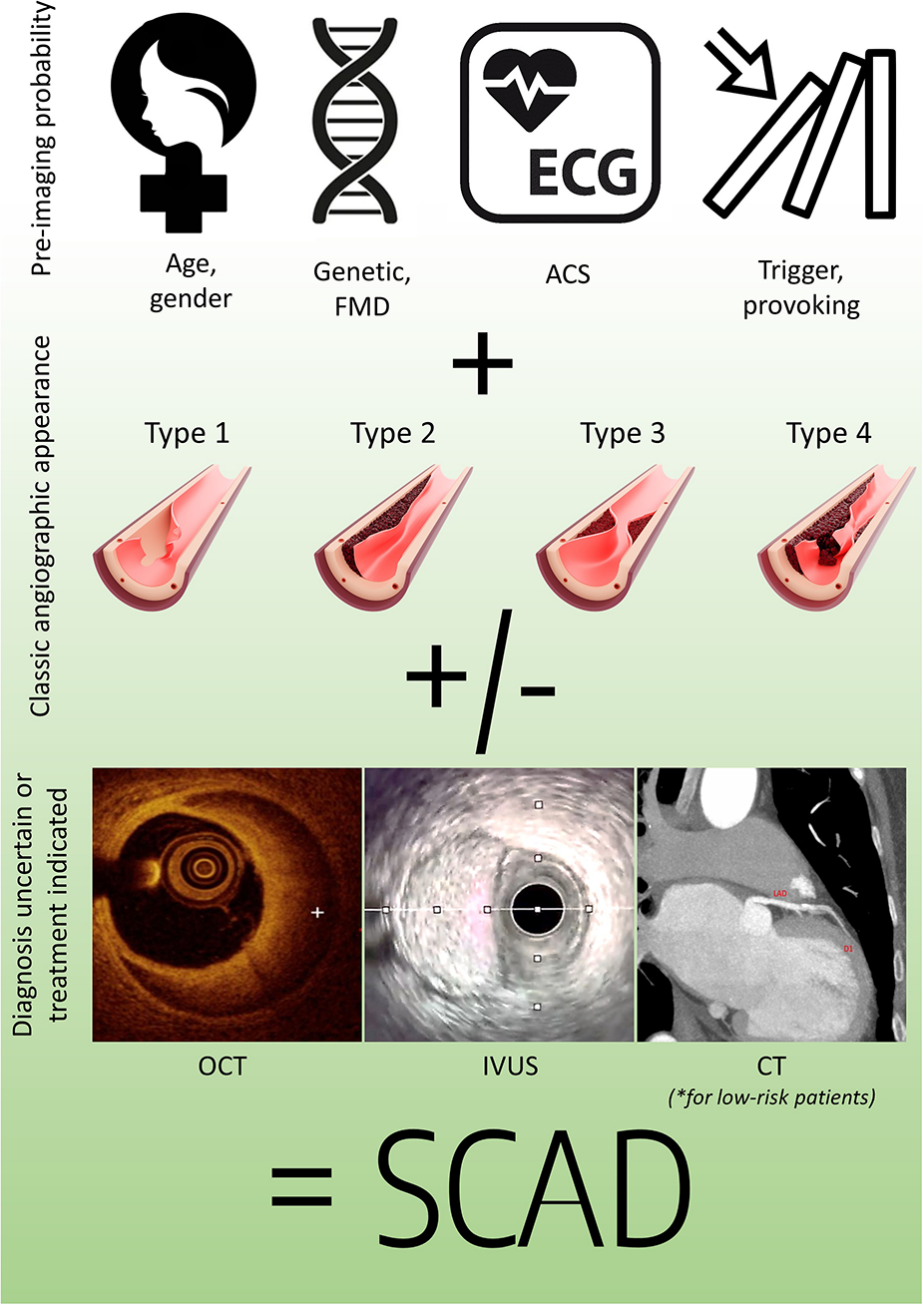
Holistic SCAD diagnostic pathway. ACS, acute coronary syndrome; CT, computed tomography; FMD, fibromuscular dysplasia; IVUS, intravascular ultrasound; OCT, optical coherence tomography.

SCAD diagnosis requires a holistic integrative approach, starting with a high level of suspicion from first-line medical professionals, due to the relatively rarity of the condition and the potential for its symptoms to mimic those of other acute conditions. Since SCAD can be life-threatening, early and accurate diagnosis is crucial for providing appropriate and timely treatment. The comprehensive diagnostic approach for SCAD begins with an assessment of preimaging probability, considering the patient's medical history, risk factors, and presenting symptoms that can streamline further diagnostic tests. By incorporating findings from various stepwise multimodality imaging techniques that can confirm possible disruption of the coronary wall integrity, treating practitioners can increase the certainty of diagnosing this relatively rare phenomenon, and, most importantly, this enables them to make informed decisions regarding further treatment strategies (Figure 5) (2, 13).

## Conclusion

SCAD presents rare but unique pathoanatomical lesion substrate among patients presenting with ACS. Proper diagnosis of SCAD begins with a high level of suspicion and awareness regarding the condition, which is subsequently supported by the utilization of non-invasive and invasive imaging modalities in order to initiate an appropriate treatment strategy. Accurate interpretation of various imaging modalities is crucial, not only for SCAD recognition, but also for deciding on subsequent treatment strategies.
